# Supplemental Nutrition Assistance Program-Education Improves Food Security Independent of Food Assistance and Program Characteristics

**DOI:** 10.3390/nu12092636

**Published:** 2020-08-29

**Authors:** Heather A. Eicher-Miller, Rebecca L. Rivera, Hanxi Sun, Yumin Zhang, Melissa K. Maulding, Angela R. Abbott

**Affiliations:** 1 Department of Nutrition Science, Purdue University, West Lafayette, IN 47907, USA; rerivera@iu.edu; 2 Richard M. Fairbanks School of Public Health and the Regenstrief Institute, Inc., Indiana University, Indianapolis, IN 46202, USA; 3 Department of Statistics, Purdue University, West Lafayette, IN 47907, USA; sun652@purdue.edu (H.S.); zhan2013@purdue.edu (Y.Z.); 4 Health and Human Sciences Cooperative Extension, Purdue University, West Lafayette, IN 47907, USA; Mkmaulding2@eiu.edu (M.K.M.); abbottar@purdue.edu (A.R.A.); 5 College of Health and Human Services, Eastern Illinois University, Charleston, IL 61920, USA

**Keywords:** supplemental nutrition assistance program-education, SNAP-Ed, nutrition education, food assistance, SNAP, food stamps, WIC, food security, food pantry, emergency food programs

## Abstract

The purpose of this project was to determine whether consistent food assistance program participation or changes in participation over time mediated or moderated the effect of federal nutrition education through the Supplemental Nutrition Assistance Program-Education (SNAP-Ed) on food security and determine the associations of SNAP-Ed program delivery characteristics with change in food security. This secondary analysis used data from a randomized controlled trial from September 2013 through April 2015. SNAP-Ed-eligible participants (*n* = 328; ≥18 years) in households with children were recruited from 39 counties in Indiana, USA. The dependent variable was one year change in household food security score measured using the United States Household Food Security Survey Module. Assessment of mediation used Barron-Kenny analysis and moderation used interactions of food assistance program use and changes over time with treatment group in general linear regression modeling. Program delivery characteristics were investigated using mixed linear regression modeling. Results showed that neither consistent participation nor changes in food assistance program participation over time mediated nor moderated the effect of SNAP-Ed on food security and neither were SNAP-Ed program delivery characteristics associated with change in food security over the one year study period. SNAP-Ed directly improved food security among SNAP-Ed-eligible Indiana households with children regardless of food assistance program participation and changes over time or varying program delivery characteristics.

## 1. Introduction

Members of low-income households face a high burden of food insecurity, poor nutrition, and undesirable health outcomes [1,2,3,4,5]. The Supplemental Nutrition Assistance Program-Education (SNAP-Ed) is a program of the United States Department of Agriculture (USDA) Food and Nutrition Service (FNS) that offers education on nutrition, budgeting, and resource management to low-income households to improve dietary intake and food security [6,7]. SNAP-Ed has been shown to improve household and adult food security in previous longitudinal randomized controlled trials [8,9]. Approximately 73% of households interested in receiving SNAP-Ed also report participating in at least one of three other food assistance programs [9] directed to alleviate food insecurity in qualifying low-income households [10], including the Supplemental Nutrition Assistance Program (SNAP), the Special Supplemental Nutrition Program for Women, Infants, and Children (WIC), and The Emergency Food Assistance Program (TEFAP). SNAP and WIC provide financial and food resources to help individuals and families obtain foods to supplement their nutritional needs [11,12] while TEFAP provides foods to state agencies who partner with private and local organizations to distribute emergency foods to food banks and food pantries where individuals in need may access foods at no cost [13]. Mutual participation in SNAP-Ed and SNAP is not required; some SNAP-Ed participants may not qualify for SNAP benefits or choose not to participate in SNAP. Further, sometimes SNAP-Ed lessons are used to fulfill WIC education requirements.

Previous evidence of improvement in food security because of SNAP [2], WIC [14], and associations with emergency program use [15], taken with knowledge of the common practice of simultaneous participation in food assistance programs and nutrition education programs, suggests that the changes observed in food security previously attributed to nutrition education [9] may actually be accounted for by participation or change in participation of food assistance (mediation). It may also be likely that the effect of nutrition education on food security may be differential by food assistance participation or changes in food assistance (moderation). SNAP-Ed educators commonly help participants with eligibility and encourage their application for local, state, and federal food assistance as part of the resource management education offered, making salient the reality that participation status in food assistance programs may frequently change during nutrition education participation [16]. Previous investigation of nutrition education program effectiveness on food insecurity has focused on singular program use and has not considered mediation or moderation by food assistance participation or changes in their use, specifically regarding the three most common food assistance programs, SNAP, WIC, and TEFAP [17]. Only one previous non-experimental short-term study evaluated joint use of two of these programs and showed that SNAP-Ed participants who were also receiving SNAP benefits and made more improvement in resource management skills, reported the greatest decrease in running out of food (measured by only one question) compared with participants who were not receiving SNAP benefits and who had less improvement in resource management skills [18]. Additional factors of relevance in SNAP-Ed effect on participant food security improvement are SNAP-Ed program delivery characteristics, such as the number of lessons, group or individual lessons, or SNAP-Ed educator. In Indiana, over sixty educators deliver up to ten SNAP-Ed lessons using group and individual lesson delivery. Program variability presented by these characteristics are inherent to SNAP-Ed and may potentially be associated with an effect on food security. For example, food security improvement may be influenced by participants receiving 10 rather than 4 lessons, individualized compared with group lessons, or by interaction with a particular SNAP-Ed educator.

Therefore, determining the potential mediating or moderating role of food assistance participation and changes in participation over time on nutrition education program participation would clarify knowledge of impacts to food security. Examination of the role of SNAP-Ed program characteristics number of lessons, delivery format, and variability of educator to food security improvement would inform program and policy of important programmatic aspects of success. The objectives of this paper were investigated among adults ≥18 years from Indiana in a dataset where a decrease of 1.2 ± 0.4 (mean ± SE) units in household food security score over the one year study period, indicating a meaningful longitudinal improvement in food security among the intervention compared to the control group, was previously discovered [9], and included:

1. Determine whether participation and changes in participation status in food assistance programs SNAP, WIC, and food pantries over one year mediated the effect of a SNAP-Ed intervention on one year change in household food security.
2. Determine whether participation and changes in participation status in food assistance programs SNAP, WIC, and food pantries over one year moderated the effect of a SNAP-Ed intervention on one year change in household food security.
3. Determine whether the number of SNAP-Ed lessons received as an intervention, SNAP-Ed lesson delivery format, or variability of SNAP-Ed educator was associated with one year change in household food security (independent of food assistance program participation).

## 2. Materials and Methods

### 2.1. Study Population

For this secondary data analysis, all data were obtained from The Indiana SNAP-Ed Long-term Study, a longitudinal (one year) parallel-arm randomized controlled nutrition education intervention trial conducted between August 2013 and April 2015 [9]. Thirty-five county-level Indiana SNAP-Ed nutrition education paraprofessionals (SNAP-Ed educators) recruited adult participants (*n* = 575) aged ≥18 years from August 2013 to March 2014 and administered baseline assessments. Participants were recruited from locations such as WIC clinics, food pantries, or Indiana Cooperative Extension county offices. The one year follow-up assessments were completed from September 2014 through April 2015. Data to address the hypotheses of this study are expected to maintain relevance to current program and participants as food insecurity in Indiana from 2013–2015 was not statistically significantly different from 2016–2018 estimates [5], and the data represent a unique opportunity to comprehensively address hypotheses using a singular sample. Only participants who completed the study (i.e., baseline and one year follow-up assessments) were included in the analysis presented here (total *n* = 328, control *n* = 163, intervention *n* = 165). SNAP-Ed educators were trained to determine participant study eligibility and randomly assigned participants to either the non-active control group or intervention group using an allocation ratio of ~1:1. A random number allocated the first participant or group recruited simultaneously (to prevent knowledge of different treatment) to the intervention or control group and then an alternating assignment was followed. After treatment group assignment, SNAP-Ed educators delivered lessons to the intervention group participants as per program protocol over the following four to ten weeks, at approximately 1 lesson per week, and facilitated all survey assessments to both treatment groups. Eligible study participants included Indiana adult residents who had one or more children living in the household, had not received a SNAP-Ed lesson in the past one year, were able to speak, read, and write in English, and were willing to wait one year to receive nutrition education lessons.

### 2.2. Intervention

The intervention consisted of the first four (out of ten) lessons in the Indiana SNAP-Ed curriculum [19] as these lessons comprise SNAP-Ed guidance and cover the USDA key behavioral outcomes of maintaining caloric balance over time for a healthy weight and consumption of nutrient-dense foods and beverages. Additionally, lessons included instruction on budgeting food resources through the following lesson topics: applying USDA MyPlate to build healthy meals, using food labels to make healthy choices, identifying the importance of whole grains, and adding more fruits and vegetables to meals [19,20]. The Purdue Institutional Review Board approved the trial protocol and all participants provided written informed consent. The trial was registered at www.clinicaltrials.gov as NCT03436589.

### 2.3. Food Security Measures

Household food security score was measured using the 18-item USDA U.S. Household Food Security Survey Module (US HFSSM) with scores ranging from 0 (food secure) to 18 (very low food secure) and a 12-month reference period [21,22]. Categorical classification of food security at baseline was also constructed as food secure, marginally food secure, and food insecure according to prior guidance [22]. Change in food security score was the response variable in this secondary data analysis to determine a more specific change compared with using food security categories, and was quantified by subtracting the baseline score from the one year follow-up score for each participant.

### 2.4. Food Assistance Program Measures Used in Objectives 1 and 2

Study participants self-reported participation status in SNAP, WIC, and food pantries over the 30 days prior to both baseline and one year follow-up assessments because the food assistance provided through these programs are generally distributed on a monthly basis. One month or 30 days was considered the minimal amount of time that these programs may exert influence on a participant household and on SNAP-Ed effectiveness. Missing values were 8% (*n* = 27) at baseline and 15% (*n* = 50) at follow-up. A sensitivity analysis was conducted where missing values were coded as participation and compared to coding values as non-participation. The results did not change so coding as non-participation was applied. Participation in local, state, or national food assistance programs other than SNAP, WIC, or food pantries was not recorded.

Three individual four-level categorical variables referred to as “change in one year participation status” were created for SNAP, WIC, and food pantries, respectively, to represent any changes or no changes in food assistance participation status between the 30 days prior to baseline and the 30 days prior to one year follow-up assessments. “Change in one year participation status” variables were created by concatenating the baseline and one year follow-up binary variables to simultaneously represent the participation status for each of the food assistance programs at baseline and at one year follow-up in addition to change in participation status if it occurred (00 = no participation; 10 = participation at baseline only; 01 = participation at one year follow-up only; 11 = participation at both baseline and one year follow-up). These variables were used as a categorical independent variable to address the first and second research objectives, whether change in food assistance program participation status or consistency mediated or moderated the impact of SNAP-Ed on one year change in food security score.

### 2.5. SNAP-Ed Program Characteristics Measures Used in Objective 3

The number of SNAP-Ed lessons a participant received, the lesson delivery format, and which SNAP-Ed educator delivered the lessons were investigated as the SNAP-Ed program characteristics among intervention group participants who completed the required four lessons to address the third research objective. Participants assigned to the intervention group that did not complete the four required intervention lessons, lost contact with SNAP-Ed educators, or did not follow the study protocol were considered withdrawn from the study (*n* = 87). The number of lessons (4–10 lessons) a participant received was recorded by the SNAP-Ed educator at each lesson and summed at the one year follow-up assessment. Lesson delivery format was a categorical variable with three levels representing how the participant received lessons (one-to-one lessons, group lessons, combination of one-to-one and group lessons) and was based on the preference of the participant to attend group lessons, educator facilitation, and the schedule of group or individual lessons. Assignment of SNAP-Ed educator (*n* = 37) was determined by the participant’s county of residence at recruitment.

### 2.6. Other Covariates

A binary variable for treatment group (control, intervention) was used to address the first and second research objectives. Time was included as a binary variable in mixed regression modeling (baseline, follow-up) to address the third research objective. Self-reported baseline participant characteristics identified as potential confounders through Chi-square comparisons between the intervention and control groups were investigated: sex (female, male); age in years (18–30, 31–50, ≥51); race/ethnicity (non-Hispanic white, other); highest level of education among the household (no high school diploma, high school diploma, or General Educational Development certification indicating high school level skills; some college/associate’s degree; ≥bachelor’s degree); marital status (living with partner/married, never married, divorced/separated/widowed); household employment (household member employed, no household member employed); household poverty status (<federal poverty guideline, ≥federal poverty guideline); household size (two, three, four, or ≥five household members); SNAP, WIC, or food pantry participation status 30 days prior to baseline (not participating, participating), and food security category at baseline (food secure, marginally food secure, food insecure). Two categories for race/ethnicity were used in this study because reports other than non-Hispanic white were very few: 3 participants reported American Indian, 1 reported Asian, and 7 reported non-Hispanic black. Maintaining separate categories would threaten the robustness of the analysis and model fit so categories were combined to a single category.

### 2.7. Statistical Methods

**Figure 1:**
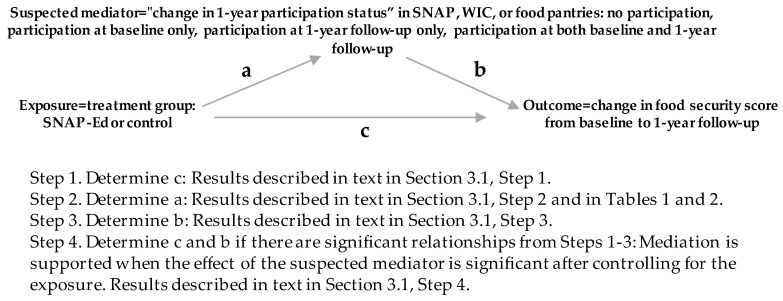
Hypothesized Baron-Kenny causal mediation model of the Supplemental Nutrition Assistance Program-Education (SNAP-Ed) intervention effect by the “change in one year participation status” in Supplemental Nutrition Assistance Program (SNAP), Women, Infants, and Children (WIC), or food pantries on the change in one year food security score among Indiana SNAP-Ed Study participants. a = the relationship of the exposure on the suspected mediator using regression, b = the relationship of the suspected mediator on the outcome using regression, c = the relationship of the exposure on the outcome using regression.

To address the first research objective, the Baron-Kenny causal mediation approach was used to investigate whether the suspected mediator “change in one year participation status” in SNAP, WIC, or food pantries mediated the effect of the exposure, SNAP-Ed intervention, on the outcome, change in household food security score over the one year study period [23]. Additional covariates are not included in the Baron-Kenny three variable system regression approach (Figure 1, below); investigation of the role of other covariates are outside of the scope of the hypotheses of this paper.

To address the second research objective, interactions between “change in one year participation status” (SNAP, WIC, and food pantries) and treatment group variables were used in general linear regression modeling to determine whether the change in food assistance program participation, consistent participation, or non-participation moderated the effect of SNAP-Ed on the change in food security score over the one year study period. SNAP, WIC, and food pantry interactions with treatment group were investigated in separate models; the reference group was consistent non-participation during the 30 days prior to baseline and one year follow-up. Other participant characteristics (sex, age, race/ethnicity, education, poverty status, employment status, marital status, household size) were initially included in the models as potential confounders but removed because they were not influential (*p* < 0.2). Statistical power to detect a difference at a significance level of α = 0.05 with power at 0.90, for a one unit improvement in food security based on previous study data [9,18,24] was confirmed using a power analysis procedure for general linear regression models. A treatment effect of one unit on the food security scale was chosen for the power analysis because of the practical relevance and potential of a one unit decrease to transition a participant between two food security statuses and the associated positive benefit. In addition, an approximate one unit change was discovered in the study from which this data was derived and considered reasonable. Tukey adjustment for multiple comparisons was applied.

To address the third research objective, a mixed linear regression model was used to determine the association of the number of lessons, lesson delivery format, and variability between SNAP-Ed educators with change in food security score over one year among the intervention group (*n* = 165). Time, number of lessons, and lesson delivery format were included as fixed effects in the model. Participants and SNAP-Ed educator were considered random effects. The covariance structure was specified as compound symmetry after using the Sawa Bayesian information criterion (BIC) to compare various covariance structures. None of the potential participant characteristic confounders were found influential (*p* > 0.2), except for age (*p* = 0.02) which was included as a covariate in the model. Statistical power to detect a difference at a significance level of α = 0.05 with power at 0.90 and one unit improvement in food security was confirmed using a power analysis procedure for mixed linear regression models.

Model assumptions were checked by plotting residuals against predicted means, Q-Q plots, and histograms of residuals for general and mixed linear regression modeling and applied to each study objective. All analyses were completed using SAS^®^ software version 9.4 (SAS Institute Inc., Cary, NC, USA).

## 3. Results

The characteristics and food security of participants in the intervention and control groups are shown in Table 1.

Participation in WIC, food pantries, and employment were the only characteristics with significantly different distributions among intervention and control groups at baseline.

### 3.1. Research Objective 1: Test for Food Assistance Program Mediation of SNAP-Ed Effect on Food Security

Step 1: Food security score did not differ between treatment groups at baseline using regression (β = −0.4, SE = 0.3, *p* = 0.4). The SNAP-Ed treatment group exposure had a significantly improved food security change from baseline to 12 months later (β = 1.2, SE = 0.4, *p* = 0.001).

Step 2: Participation status in WIC and food pantry use, but not for SNAP, 30 days prior to baseline differed (*p* < 0.01) between the intervention and control groups in Chi-square analyses (Table 1). Additionally, “change in one year participation status” (30 days prior to baseline and one year follow-up) in WIC and food pantry use differed (*p* = 0.03) between the intervention and control groups using Chi-square analysis (Table 2), but again, not for SNAP (*p* = 0.3). Logistic regression showed similar results of an association with treatment group and the potential for mediation for WIC (*p* = 0.04) and food pantry use (*p* = 0.05) but not SNAP (*p* = 0.3) (Table 2).

Step 3: Using general linear regression modeling, “change in one year participation status” in SNAP (*p* = 0.3), WIC (*p* = 0.4), or food pantry use (*p* = 0.5) were not associated with the long-term change in food security score.

Step 4: Since significant relationships were present in steps 1 and 2, multiple linear regression modeling of the relationship of treatment group and “change in one year participation status” in SNAP, WIC, and food pantries on the outcome was completed. Results showed that neither SNAP (*p* = 0.2), WIC (*p* = 0.2), nor food pantries (*p* = 0.3) were significant after treatment group was included in the model, yet treatment group remained significant (*p* ≤ 0.001).

In conclusion of research objective 1, no mediation was found between the SNAP-Ed intervention and “change in one year participation status” in SNAP, WIC, or food pantries on the change in food security score over the one year study period in the intervention compared to the control group using the Baron-Kenny causal mediation approach.

### 3.2. Research Objective 2: Test for Food Assistance Program Moderation of SNAP-Ed Effect on Food Security

The interactions of “change in one year participation status” in SNAP, WIC, or food pantries with the treatment group did not moderate the mean difference (mean ± SEM) in food security scores in the intervention compared to the control over the one year study period using general linear regression modeling (SNAP −0.8 ± 0.4, *p* = 0.2; WIC −1.1 ± 0.5, *p* = 0.1; food pantries −1.2 ± 0.8, *p* = 0.7) (Table 3).

### 3.3. Research Objective 3: Test for SNAP-Ed Program Characteristics Relationship with SNAP-Ed on Food Security

The majority of intervention group participants (*n* = 165, 78%) received more than the minimum of four lessons with a mean of 6.8 lessons (Table 4). Approximately half of participants (*n* = 85, 57%) received lessons in a one-to-one or individualized format, followed by group (*n* = 38, 26%), and combination the two types (*n* = 25, 17%). There was no statistical evidence of an association between lesson delivery format (*p* = 0.3), the number of lessons received (*p* = 0.6), or variation between SNAP-Ed educators (*p* = 0.4) and the mean increase in food security score over time using a mixed multiple linear regression model.

## 4. Discussion

The major finding from this secondary data analysis indicated an improvement in household food security among the SNAP-Ed intervention group compared to the control group regardless of participation and changes in participation in food assistance programs SNAP, WIC, or food pantries 30 days prior to baseline and one year after the intervention. The mediation and moderation analyses addressing research objectives one and two revealed that SNAP-Ed directly improved food security rather than exerting or magnifying improvement through food assistance participation or changes in participation over one year.

One previous study found greater improvements in food security among SNAP-Ed participants who also received SNAP [18] indicating that for certain populations and shorter time periods, SNAP may assist SNAP-Ed to further improve food security. However, the present results using experimental data, determined no significant difference between the treatment groups for change in food security across the four types of one year SNAP participation status. Together, previous and current study results build evidence that SNAP-Ed is effective in directly improving food security over a one year period [9].

In addition to improving food security, SNAP-Ed may have caused changes in participation status in food assistance programs throughout the study period for the following reasons. As part of the normal program delivery, SNAP-Ed educators may have encouraged and assisted intervention group participants who were not receiving food assistance at baseline to apply for financial benefits through SNAP or WIC or to maximize nutrition resources available through food pantries or other resources. On the other hand, improvements in food security directly from SNAP-Ed may have led intervention group participants who reported receiving food assistance at baseline to attain and maintain sufficient nutrition resources and withdraw participation in SNAP, WIC, or use of food pantries by the one year follow-up. Alternatively, participation in other local, state, or federal food assistance programs or resources that were not recorded in this study may have impacted food security. For example, policy, systems, environment, and other nutrition and lifestyle related resources may be influential in the success of SNAP-Ed and should continue to be investigated in the future [25]. Investigation to the reasons for changes in food assistance participation were outside of the scope of this research but present an opportunity for the future. Due to the observational nature of food assistance designation in this study, the results do not provide causal evidence of SNAP-Ed influence on changes in food assistance participation status. This limitation provides an important research opportunity, yet ethical constraints may hinder randomization of food assistance resources and require pragmatic study designs in future research [18].

In addition to finding no mediation or moderation of changes or consistency in food assistance program participation on SNAP-Ed effectiveness on food security, nutrition education program characteristics such as the number of lessons, delivery format (group or individual lessons), and SNAP-Ed educator were not associated with the magnitude of SNAP-Ed effectiveness on food security. A study describing the effect of online compared to in-person SNAP-Ed lesson delivery [26], on nutrition knowledge, intentions to change behavior, and self-efficacy, is the only previously published SNAP-Ed study to evaluate similar SNAP-Ed program characteristics. No previously published studies have addressed the question of a dose-response effect of the number of SNAP-Ed lessons on food security. In the study described herein, more than four lessons did not result in a significantly larger improvement in food security. The minimum lessons comprising SNAP-Ed guidance, four in this case, were a sufficient intervention to improve food security, reinforcing the notion that these limited lessons cover the most important behavioral recommendations for SNAP-Ed set by the USDA FNS at least in regard to food security [21]. The results suggest that participation in the minimally adherent intervention lessons is more critical to food security gains than the frequency and amount of additional time spent in lessons. Other beneficial outcomes that were not quantified here, such as sustainability of food security gains over a period longer than one year, increased nutrition knowledge, or dietary changes, may potentially be influenced by additional lessons; however, those outcomes have yet to be investigated.

The format of lesson delivery was also not significantly associated with change in food security over the one year study period among the intervention group. A current Indiana SNAP-Ed priority set forth by the USDA FNS encourages a transition to mostly group lesson delivery format rather than one-to-one format. This policy decision is supported by these study results in regard to food security improvements. Group lessons reach a greater number of participants at less cost and time, and, in this study, were as effective as individual lessons. Yet, reach to participants with special needs was not evaluated here and the provision of individual lessons may remain relevant for this group.

The third program characteristic assessed in this study, variability in one year food security score due to different educators, was not statistically significant. Variable characteristics inherent to the educator that may potentially influence outcomes include age, race, ethnicity, language, gender, education level, years of experience, depth of nutrition education knowledge, personality, knowledge and connection with community resources, among many others. These characteristics may affect the delivery and acceptance of the program to participants by potentially influencing SNAP-Ed educators’ and participants’ abilities to connect and relate to each other. Investigating the educator as a random effect in the model did not allow for comparisons specifically based on the educator characteristics mentioned or between specific educators yet, did allow insight to educator significance with regard to SNAP-Ed effectiveness. The study results suggest that the SNAP-Ed educators delivered a program effective at improving participants’ household food security irrespective of educator.

A few studies have evaluated the impact of SNAP-Ed on food security; however, there is a paucity of SNAP-Ed literature specifically evaluating the impact of program delivery characteristics on food security outcomes [8,9,18]. A small body of literature has evaluated a second federally-supported nutrition education program, the Expanded Food and Nutrition Education Program (EFNEP) [27,28,29,30]. Since the two programs are similar in terms of aligning program goals with the Dietary Guidelines for Americans and target population, research results from EFNEP provide relevant background. Studies evaluating EFNEP reported an increase in food security using a variety of food security measures including one survey question [27] and the 6-item [28] and 18-item [30] US HFSSM. The number of lessons needed to increase food security greatly varied across the studies. In one study, program completers (mean number of lessons 8.5 ± 0.02) compared to drop-outs (mean number of lessons 6.8 ± 0.11) showed a positive dose-response in food security with increasing number of lessons [27]. Additionally, food security was higher in participants who received lessons in a one-to-one format compared to those who received lessons in a group format or a combination of group and individual lessons [27]. In other studies, participants improved food security after receiving seven EFNEP lessons [28] or with just two or more lessons compared to a comparison group receiving one or no lessons [30]. Lesson delivery format was not always defined in these studies. The results of the present study strengthen the evidence that effectiveness of nutrition education to improve food security does not depend on the number of lessons exceeding the program completion criteria, nor format of lessons (group or one-to-one), despite the mixed results from the small body of EFNEP and SNAP-Ed literature.

Results from the present study provide a foundation for further research that improves upon some limitations, but others are presented. Treatment groups were not originally designed to test participation in singular or concurrent food assistance programs or program characteristics as main effects in the analysis. The implication of the simple randomization technique in conjunction with the large number of potential confounding characteristics presents a possibility for uneven distribution of characteristics across treatment groups, which could result in overestimation their effects. Although no significant effect was detected in this study, designing future studies to further stratify the control and intervention groups by food assistance participation status may enhance evaluation of simultaneous food assistance program participation and changes in participation and nutrition education on target outcomes. Potential for misclassification was present; however, non-response was low (baseline 8% (*n* = 27), follow-up 15% (*n* = 50)) and did not influence the results based on the sensitivity analysis, but the hesitation for some participants to answer these types of sensitive survey questions is important to consider when calculating future study sample sizes and mitigation of bias. Specifically, responses on the HFSSM were made for the entire household by one adult in the household (as per guidance [22]) and entail the reporting adult’s perceptions on the other household member’s food security. The 30-day reference periods before baseline and one year follow-up may not have captured all changes in food assistance. Collecting additional information on the consistency and timing of food assistance use in future studies could elucidate the temporality of the relationship between food assistance program participation, SNAP-Ed, and food security improvement. Interpretation of the results should be carefully limited to the hypothesis focused on SNAP-Ed as the main independent variable and do not inform the role of SNAP as the main independent variable on food security status.

A major strength of this study was the use of longitudinal data derived from a randomized controlled impact evaluation showing an improvement in one year food security due to SNAP-Ed [9]. Participants included in these analyses represented the greater Indiana SNAP-Ed population except for less racial diversity (89% of Indiana SNAP-Ed participants compared to 95% of study participants were non-Hispanic White; Chi-square *p* < 0.01). This difference in racial diversity is likely due to not having SNAP-Ed educators from more racially diverse geographic areas volunteer to assist with the study. Participants who withdrew from the trial were less likely to be married or living with a partner, resided in smaller households, and reported lower incomes compared to study completers [9]. The results of this study may not be generalizable to SNAP-Ed participants who have similar characteristics as the participants who withdrew from the trial and do not classify themselves as non-Hispanic white. Quantification of the change in food security score using the US HFSSM contributed a second major strength to the study. This tool is considered to be the gold standard that is used in national surveys and other research studies, permitting comparisons of results across other populations and enhancing external validity. Use of the score allows a more specific understanding of the change in food security and relationships evaluated.

## 5. Conclusions

This study highlights nutrition education as a critical, independent component to improving food security in the US low-income population by showing SNAP-Ed directly and sustainably improves food security with or without the presence of food assistance. Neither group, individual or mixed type lessons nor SNAP-Ed educator were related to the effectiveness of SNAP-Ed on food security. Neither were provision of lessons additional to those fulfilling SNAP-Ed guidance related to the magnitude of SNAP-Ed effectiveness. The current study results, along with previous documentation of food assistance effectiveness on food security, support a need for future investigation into the longitudinal effect of participation in multiple food assistance programs, including SNAP-Ed, to maximize improvements in food security and other USDA FNS targeted health outcomes.

## Figures and Tables

**Table 1:** Comparison of baseline sociodemographic characteristics by treatment group of Indiana SNAP-Ed participants among households with children using Chi-Square analysis.

		Control		Intervention		χ^2^ *p*-Value
		*N*	%	*N*	%	
Total		163	50	165	50	
Sex						0.7
	Female	148	93	148	92	
	Male	11	7	13	8	
Age Group						0.3
	18–30 Years	77	47	93	56	
	31–50 Years	73	45	60	36	
	51 Years or Older	13	8	12	7	
Race/Ethnicity						0.7
	Non-Hispanic White	145	96	149	97	
	Other	6	4	5	3	
Household Education						0.1
	No High School Diploma	7	4	13	8	
	High School Diploma	29	18	45	27	
	General Educational Development	31	19	27	16	
	Some College	53	33	46	28	
	Associate’s Degree	23	14	25	15	
	Bachelor’s Degree or Higher	17	11	8	5	
Marital Status						0.2
	Never Married	28	17	40	24	
	Married/with partner	94	58	94	57	
	Separated/Divorced	41	25	31	19	
Household Employment						0.01 *
	Not Employed	82	50	60	36	
	Employed	81	50	105	64	
Household Poverty Status (Income to Poverty Ratio)						0.3
	≥Federal Guideline	44	27	37	22	
	<Federal Guideline	119	73	128	78	
Household Size						0.5
	2	12	7	6	4	
	3	38	23	38	23	
	4	42	26	47	28	
	5 or more	70	43	74	45	
SNAP Participation (past 30 days)						0.1
	No	76	47	62	38	
	Yes	87	53	103	62	
WIC Participation (past 30 days)						<0.01 *
	No	81	50	58	35	
	Yes	82	50	107	65	
Food Pantry Participation (past 30 days)						<0.01 *
	No	138	85	156	95	
	Yes	25	15	9	5	
Baseline Household Food Security						0.9
	Food Secure	44	27	41	25	
	Marginal	64	39	65	39	
	Food Insecure	55	34	59	36	

Values are counts, percentages, and *p*-values from Chi-square comparisons of the distributions among sociodemographic characteristics between control and intervention group participants. Total numbers do not always add to sample size due to missing values and percentages do not always add to 100 due to rounding. * *p* ≤ 0.05. Abbreviations: SNAP-Ed, Supplemental Nutrition Assistance Program-Education; SNAP, Supplemental Nutrition Assistance Program; WIC, Special Supplemental Nutrition Program for Women, Infants, and Children.

**Table 2:** Change in one year participation status comparison of SNAP, WIC, and food pantries by treatment group among Indiana SNAP-Ed participants using Chi-Square and logistic regression.

		Total		Control		Intervention		χ2 *p*-Value	Logistic Regression *p*-Value
		*n*	%	*N*	%	*n*	%		
Total		328	100	163	50	165	50		
									
	Change in One Year Participation Status								
SNAP								0.3	0.3
	No Participation	105	32	58	36	47	28		
	Baseline Participation Only	39	12	21	13	18	11		
	Follow-up Participation Only	33	10	18	11	15	9		
	Baseline and Follow-up Participation	151	46	66	40	85	52		
WIC								0.03 *	0.04 *
	No Participation	122	37	73	45	49	30		
	Baseline Participation Only	61	19	24	15	37	22		
	Follow-up Participation Only	17	5	8	5	9	6		
	Baseline and Follow-up Participation	128	39	58	35	70	42		
Food Pantry								0.03 *	0.05 *
	No Participation	278	85	130	80	148	90		
	Baseline Participation Only	18	5	13	8	5	3		
	Follow-up Participation Only	16	5	8	5	8	5		
	Baseline and Follow-up Participation	16	5	12	7	4	2		

Values are counts, percentages, and *p*-values from Chi-square and logistic regression comparisons of the distributions among “change in one year food assistance participation status” between control and intervention group participants. Total numbers do not always add to sample size due to missing values and percentages do not always add to 100 due to rounding. Reference period for one year participation status covered the 30 days prior to baseline and 30 days prior to one year follow-up. * *p* ≤ 0.05. Abbreviations: SNAP-Ed, Supplemental Nutrition Assistance Program-Education; SNAP, Supplemental Nutrition Assistance Program; WIC, Special Supplemental Nutrition Program for Women, Infants, and Children.

**Table 3:** Change in food security score over one year study period for the interaction of “change in one year participation status” and treatment group among Indiana SNAP-Ed participants using general linear regression modeling.

		Mean Change in Household Food Security Score						
		Control *n* = 163		Intervention *n* = 165		Intervention-Control		
		Mean	SE	Mean	SE	Mean Difference ^‡^	SE	*p*-Value ^§^
SNAP	SNAP × Treatment Group	−0.9	0.3	−1.7	0.3	−0.8	0.4	0.2
	No Participation	−0.8	0.4	−1.3	0.5	−0.5	0.6	1.0
	Baseline Participation Only	−2.1	0.7	−2.4	0.8	−0.3	1.0	1.0
	Follow-up Participation Only	−0.8	0.8	−1.3	0.8	−0.5	1.1	1.0
	Baseline and Follow-up Participation	0	0.4	−2.0	0.3	−2.0	0.5	<0.01
WIC	WIC × Treatment Group	−0.9	0.4	−1.9	0.3	−1.1	0.5	0.1
	No Participation	−0.6	0.4	−2.7	0.5	−2.1	0.6	<0.01
	Baseline Participation Only	−1.0	0.7	−0.8	0.5	0.2	0.8	1.0
	Follow-up Participation Only	−1.5	1.1	−2.7	1.1	−1.2	1.6	1.0
	Baseline and Follow-up Participation	−0.4	0.4	−1.5	0.4	−1.1	0.6	0.5
Food Pantry	Food Pantry × Treatment Group	−0.9	0.4	−2.1	0.6	−1.2	0.8	0.7
	No Participation	−0.5	0.3	−1.8	0.3	−1.3	0.4	0.03
	Baseline Participation Only	−0.4	0.9	−3.2	1.5	−2.8	1.7	0.7
	Follow-up Participation Only	−0.6	1.1	−0.9	1.1	−0.3	1.6	1.0
	Baseline and Follow-up Participation	−2.3	0.9	−2.8	1.6	−0.5	1.9	1.0

Least squares means were calculated using general linear regression models with change in food security as the response variable. SNAP, WIC, and food pantries were investigated in separate models including interactions with treatment group. ^‡^ A decrease in food security score from baseline to 1 year follow-up indicates improved food security. ^§^ Tukey adjustment for multiple comparisons in stratified analyses in each model. Interactions of each food assistance program with treatment were significant when interaction term *p* ≤ 0.05. Abbreviations: SE, Standard Error of the Least Squares Mean; SNAP-Ed, Supplemental Nutrition Assistance Program-Education; SNAP, Supplemental Nutrition Assistance Program; WIC Special Supplemental Nutrition Program for Women, Infants, and Children.

**Table 4:** Evaluation of lesson delivery format, SNAP-Ed educator, and number of lessons received by Indiana SNAP-Ed Study participants on change in food security score over one year study period using mixed multiple linear regression modeling.

	Control Group		Intervention Group		
Program Characteristic	*N*	%	*n*	%	*p*-Value ^§^
Total	163	50	165	50	
Lesson Delivery Format					0.3
Individual	-	-	85	57	
Group	-	-	38	26	
Combination	-	-	25	17	
Number of Lessons					0.6
0	163	100	-	-	
4	-	-	37	22	
5	-	-	25	15	
6	-	-	25	15	
7	-	-	9	6	
8	-	-	12	7	
9	-	-	23	14	
10	-	-	34	21	
SNAP-Ed Educator					0.4

Lesson delivery format was reported at baseline assessment. Number of lessons was reported at the one year follow-up assessment. The control group did not receive lessons. A minimum of 4 lessons was required to have completed the intervention. Only treatment group participants were included in the mixed multiple linear regression modeling. Cells do not always add to total sample size due to missing data. ^§^ *p*-values reported for lesson delivery format and number of lessons are from the type 3 test of fixed effects. The *p*-value reported for SNAP-Ed educator is from the random effect covariance parameter estimate. Abbreviations: SNAP-Ed, Supplemental Nutrition Assistance Program-Education.
